# The Effect of Anti-TNF Therapy on Cardiac Function in Rheumatoid Arthritis: An Observational Study

**DOI:** 10.3390/jcm9103145

**Published:** 2020-09-29

**Authors:** Milad Baniaamam, M. Louis Handoko, Rabia Agca, Sjoerd C. Heslinga, Thelma C. Konings, Vokko P. van Halm, Mike T. Nurmohamed

**Affiliations:** 1 Amsterdam Rheumatology Immunology Center, Location Reade, 1056 AB Amsterdam, The Netherlands; r.agca@reade.nl (R.A.); s.heslinga@amsterdamumc.nl (S.C.H.); m.nurmohamed@reade.nl (M.T.N.); 2 Amsterdam Cardiovascular Sciences, Vrije Universiteit, 1081 HZ Amsterdam, The Netherlands; 3 Department of Cardiology, Amsterdam UMC, Vrije Universiteit Amsterdam, 1081 HV Amsterdam, The Netherlands; ml.handoko@amsterdamumc.nl (M.L.H.); t.konings@amsterdamumc.nl (T.C.K.); v.p.vanhalm@amsterdamumc.nl (V.P.v.H.); 4 Department of Rheumatology, Amsterdam UMC, Vrije Universiteit Amsterdam, 1081 HV Amsterdam, The Netherlands

**Keywords:** rheumatoid arthritis, inflammation, anti-TNF, cardiac function, echocardiography

## Abstract

Congestive heart failure (CHF) is the second most prevalent cause of death in rheumatoid arthritis (RA). The systemic inflammatory state in RA patients is deemed responsible for this finding. Anti-inflammatory treatment with anti-tumor necrosis factor (anti-TNF) therapy decreases CV risk and subsequently might improve the cardiac function by lowering the overall inflammatory state. This study investigated the effect of anti-TNF on the cardiac function in RA patients. Fifty one RA patients were included, of which thirty three completed follow-up. Included patients were >18 years, had moderate–high disease activity and no history of cardiac disease. Patients were assessed at baseline and after six months of anti-TNF treatment. Patients underwent conventional Speckle tracking and tissue Doppler echocardiography in combination with clinical and laboratory assessments at baseline and follow-up. The left ventricular ejection fraction (LVEF) and global longitudinal strain (GLS) showed no changes during follow-up, LVEF 63% (±9) to 62% (±8) *p* = 0.097 and GLS −20 (±4) to −20 (±3) *p* = 0.79, respectively. Furthermore, E/e’ nor E/A changed significantly between baseline and follow-up, respectively 8 (7–9) and 8 (7–9) *p* = 0.17 and 1.1 (±0.4) and 1.1 (±0.4) *p* = 0.94. Follow-up NT-proBNP decreased with 23%, from 89 ng/L (47–142) to 69 ng/L (42–155), *p* = 0.10. Regression analysis revealed no association between change in inflammatory variables and cardiac function. Echocardiography showed no effect of anti-TNF treatment on the cardiac function in RA patients with low prevalence of cardiac dysfunction. Moreover, NT-proBNP decreased, possibly indicating (subtle) improvement of the cardiac function.

## 1. Introduction

Patients with rheumatoid arthritis (RA) have a 1.5-fold increased risk of cardiovascular (CV) mortality [1,2]. The systemic inflammatory state in RA patients is deemed responsible for this increased risk by causing endothelial dysfunction and accelerating atherosclerosis [3,4]. Second after myocardial infarction, congestive heart failure (CHF) is one of the most prevalent causes of death in RA patients [5,6]. The damage caused by a myocardial infarction and formation of subsequent scar tissue might be a cause of the elevated incidence of heart failure. However, a direct effect of the systemic inflammation itself has also been suggested as a cause for the development of left ventricular (LV) dysfunction. The latter is possibly explained by a process in which circulating pro-inflammatory mediators, such as tumor necrosis factor (TNF), induce coronary endothelial activation leading to stiffness of the myocardium and interstitial fibrosis deposition, resulting in impairment of the relaxation of the myocardium (diastolic dysfunction) [7]. Particularly, RA studies have shown an increased prevalence of left ventricular diastolic dysfunction [8,9,10]. This explanation is underlined by studies demonstrating that the increased incidence of CHF is only partly due to increased prevalence of CV risk factors, such as hypertension, dyslipidemia and increased insulin resistance. However, even after correction for these traditional risk factors, the increased risk for CHF remains [11,12].

Several studies have shown that anti-inflammatory treatment with anti-TNF therapy decreases the CV risk in RA [13,14,15]. It is plausible that anti-TNF therapy improves the cardiac function by lowering the overall inflammatory state by decreasing coronary endothelial activation and slowing down the process of coronary atherosclerosis [16], thus potentially decreasing the risk of developing CHF in RA patients.

However, previous studies assessing the effect of anti-TNF on the cardiac function have shown conflicting results [17,18]. Firstly, anti-TNF therapy is contra-indicated in (RA) patients with CHF (New York Heart Association class III and IV) [18], following trials from the early ‘00s suggesting that anti-TNF possibly worsens CHF and increases mortality in non-RA patients with systolic CHF. It must be recognized, however, that the interpretation of these results is still controversial to date [18]. Moreover, several trials did not show a detrimental effect of anti-TNF therapy on the incidence of newly onset CHF in RA patients [19,20]. Two imaging studies assessing the effect of infliximab on the cardiac function with echocardiography showed improved measures of systolic and diastolic function, subsequently an increased left ventricular ejection fraction (LVEF) and a decrease of E/e’ (parameter for diastolic function) after at least 3 months treatment, suggesting improvement of the systolic as well as diastolic function [21,22]. Finally, one study investigating the effect of anti-TNF on the cardiac function with Speckle tracking echocardiography did not show improvement of function [23]. Overall, it should be realized that these studies had relatively small sample sizes and altogether, it is presently unknown whether and to what extent TNF blocking therapy has a favorable effect on the cardiac function in patients with RA.

This study aimed to elucidate the effect of anti-inflammatory therapy, i.e., anti-TNF, on the systolic and diastolic cardiac function in RA patients assessed with comprehensive echocardiography (including conventional, tissue-Doppler and Speckle tracking) and cardiac biomarkers.

## 2. Methods

Study population—a prospective study in fifty one RA patients was performed. Subjects were recruited randomly from a large rheumatology outpatient clinic (Reade) in Amsterdam, the Netherlands and Amsterdam UMC, Vrije Universiteit Amsterdam, department of Rheumatology, the Netherlands from December 2014 and June 2018. All participants gave written informed consent and the protocol (NL49652.048.14) was approved by the medical ethics committee of the Slotervaart hospital and Reade, Amsterdam, the Netherlands. Echocardiography and clinical and laboratory assessments were done at baseline and after 6 months of anti-TNF therapy. Inclusion criteria consisted of patients fulfilling the 1987 ACR criteria for RA [24], being at least 18 years old, having moderate–high disease activity (DAS28 ≥ 3.2) or increased inflammatory biomarkers (i.e., erythrocyte sedimentation rate (ESR) > 15, C-reactive protein (CRP) > 10) and being scheduled for anti-TNF treatment. Patients started subcutaneous treatment with adalimumab (40 mg biweekly), etanercept (50 mg weekly), certolizumab pegol (400 mg biweekly during first two doses followed by 200 mg biweekly) or golimumab (50 mg monthly). Patients with a medical history of cardiac disease such as myocardial infarction and heart failure, and patients who used anti-TNF therapy 3 months prior to start of the study were excluded. Patients were assessed at baseline and after 6 months of anti-TNF treatment.

Clinical assessment—disease activity was assessed by the disease activity score DAS28 [25]. Physical examination included height, weight, blood pressure measurement and joint examination. Blood sample measurements (non-fasting) included standard hematological assessment, erythrocyte sedimentation rate (ESR), C-reactive protein (CRP), triglyceride levels, total cholesterol, high-density lipoprotein (HDL) and low-density lipoprotein (LDL), NT-proBNP and Troponin-T. All blood samples were assessed in a single laboratory. Health Assessment Questionnaire (HAQ) and patients global assessment of disease activity (VAS). Furthermore, smoking status, history and family history for cardiovascular disease (CVD) were assessed anamnestically.

**Figure 1:**
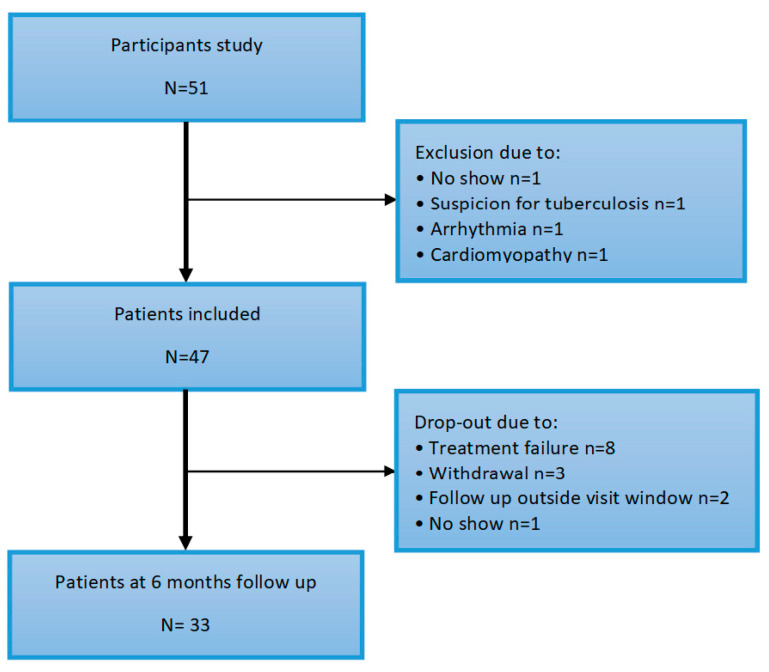
Flowchart of inclusion.

Echocardiography—transthoracic echocardiography (TTE)—was performed by certified echo technicians at the European Society of Cardiology (ESC)-certified department of echocardiography of the Amsterdam University medical center, location VUmc, using a Philips ultrasound system (Epiq 7 and IE 33, Philips, Amsterdam, NL). All echocardiographic recordings were stored digitally and were afterwards analyzed by an experienced cardiologist specialized in echocardiography (T.C.K.). TTE was performed according to the following protocol based on the guidelines provided by European Association of Echocardiography [26]. Assessment of the cardiac function consisted of apical four-, three- and two-chamber views, and 2D color and spectral flow Doppler recordings. Left ventricular mass (LVM) was computed based on the Devereux and Reichek formula [27]. Pulse wave tissue Doppler imaging was assessed in the apical views to obtain mitral annular velocities. The sample volume was located at, or within 1 cm of the septal (e’ sept) and lateral (e’ lat) mitral valve insertion sites. Doppler spectral velocity recordings of the mitral inflow were assessed with the sample volume aimed at the tips of mitral valves. From the trans mitral Doppler velocity recordings, the E wave deceleration time (DT), peak E and A velocities and the E/A ratio were acquired. Left atrial volume was obtained using the modified biplane Simpson’s rule. LVEF and diastolic and systolic volumes were computed by Simpson’s from the apical four- and two-chamber view. The left ventricular global longitudinal strain (GLS) was measured using QLab (version 10.3, Philips, Amsterdam, NL) (Figure 1). As the quality of echocardiography is subject to external factors such as excessive fat tissue, not all cardiac parameters were assessed per patient. The total number of cardiac parameter assessments are therefore described per variable in Table 1.

In addition, the modified H2FPEF score was used to calculate the a priori chance of the diastolic dysfunction in this cohort [28]. The peak TRV >2.8 m/s was suggestive for pulmonary hypertension.

Electrocardiography (ECG)—ECGs performed were standard 12-lead ECGs, recorded at 25 mm/s paper speed. ECGs were analyzed by a single investigator (T.C.K.) whom was blinded to the clinical status of all the patients.

Definitions—systolic LV dysfunction was defined as an LVEF <50%. Abnormal GLS was defined as >−17%. Diastolic function assessment was based on the ASE/EACVI 2016 guidelines [29] categorized in 4 grades: normal diastolic function and grade I-III (or indeterminate).

Statistical analysis—characteristics of the population are expressed as ±standard deviation (SD), median (interquartile range) (IQR) or percentages. For comparisons of paired continuous variables between baseline and follow-up with normal distribution, paired student’s *t*-test was used. In case of non-normal distribution, the Wilcoxon signed-ranks test or log transformation was used. For comparisons of dichotomous variables between baseline and follow-up, the Pearson’s chi-square test was performed. Regression analysis was used to assess the possible effect of change in systemic inflammation on the cardiac function with the follow-up cardiac function parameter as a dependent variable adjusted for the baseline cardiac function parameter, the baseline inflammatory parameter and delta inflammatory parameter (absolute change of the inflammatory variable at follow-up). As inflammatory parameters, the DAS28, CRP and ESR were used. LVEF, GLS, E/e’, E/A and NT-proBNP were used as values for cardiac function.

All analyses were done with SPSS version 23 (SPSS, Chicago, IL, USA) and two-sided *p*-values less than 0.05 were considered statistically significant.

The sample size was based on the primary outcome, i.e., diastolic LV function as assessed with Doppler echocardiography and was calculated using the McNemar’s test for sample size estimation. With an expected improvement of 25% in diastolic LV function during anti-TNF therapy [23,30] and a significance level of 5% and 90% power, the total calculated sample size was forty four subjects. To account for a loss to follow-up of 10%, fifty one patients were included.

## 3. Results

### 3.1. Patient Characteristics

**Figure 2:**
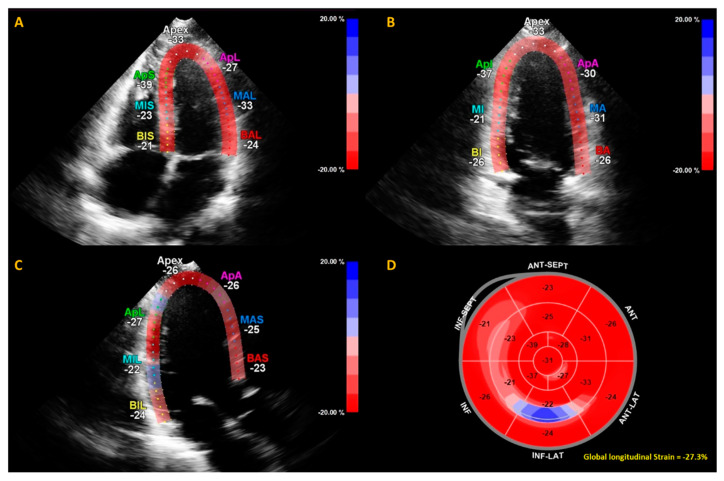
Example of calculation of global longitudinal strain (GLS) by speckle tracking for the apical four-, three- and two-chamber views (**A**–**C**). The strain during one cardiac cycle is shown for each of the color-coded (red and blue) left ventricular segments. The longitudinal strain bull’s eye plot (**D**) determined from 2D speckle tracking imaging offers a visual overview of the regional and global left ventricular myocardial function in a diagram. In this example of one of the subjects in this study, the GLS is −27.3%, which is in the normal range.

The baseline characteristics are described in Table 1. A total of fifty one subjects participated in the study. Four patients were excluded at baseline for various reasons (no show, suspicion of tuberculosis or cardiac disease de novo, arrhythmia and cardiomyopathy, depicted in Figure 2). The mean age of the patients was 57 (±11) years, of which 68% were female. Median RA disease duration was 5 (2–19) years and disease activity was moderate–high, with a mean DAS28 score of 4.44 (±1.23). Overall, the patient’s mobility was moderately impaired with a median HAQ score of 1.25 (0.75–1.50). One patient had a confirmed vascular event (ischemic CVA) in the history.

### 3.2. Cardiac Function at Baseline

At baseline, three (7%) patients had diastolic LV dysfunction, of which two (4%) had diastolic dysfunction grade I, and one (2%) had diastolic LV dysfunction grade II. Importantly, two cases of diastolic dysfunction (grade I) was due to the presence of systolic dysfunction. None of the patients with diastolic dysfunction had hypertension nor diabetes mellitus (DM). In comparison, three of the thirty eight (8%) patients with normal diastolic function had hypertension and one (3%) had DM. Systolic LV dysfunction based on the ejection fraction was seen in two patients (4%). The GLS showed impaired systolic LV function in six patients (14%).

A total of thirty seven ECGs were assessed of which three ECGs showed abnormalities. These were a left bundle branch block, a pathologic Q-wave (without a known history of myocardial infarction) and a first degree atrioventricular block.

### 3.3. Disease Activity and Cardiac Function at six Months Follow-Up

A total of thirty three patients completed follow-up. Fourteen patients dropped out of which eight due to treatment failure (lack of efficacy), three participants withdrew out of the study and of two patients fell out of the follow-up date. DAS28 decreased significantly after six months anti-TNF therapy, from 4.44 (±1.23) to 2.72 (±1.23), *p* < 0.001. Furthermore, on average the patients mobility as scored with the HAQ improved from 1.3 (0.8–1.5) to 0.5 (0.0–1.3), *p* = 0.001. LVEF and GLS showed no change after 6 months of anti-TNF therapy, respectively 63.0% (±8.7) to 62.0% (±7.9), *p* = 0.097 and −19.8 (±3.5) to -19.9 (±2.6), *p* = 0.79. See Table 2.

From thirty of the thirty three patients with successful follow-up, it was possible to grade the diastolic function. Of these patients, twenty eight had normal diastolic function, one had diastolic dysfunction grade I and one diastolic dysfunction grade II. No patients had diastolic dysfunction grade III. Compared to the baseline, only in one case was the diastolic function changed from normal to grade I. Additionally, neither the E/e’ nor the E/A showed a significant change after six months of anti-TNF therapy, respectively 7.9 (6.6–9.0) to 7.7 (7.1–9.1), *p* = 0.17 and 1.1 (±0.4) to 1.1 (±0.4), *p* = 0.94.

NT-proBNP values decreased, although this did not reach statistical significance, with a reduction of 23% at follow-up compared to baseline, respectively 89 ng/L (47–142) to 69 ng/L (42–155, *p* = 0.10). Troponin-T did not show any change at follow-up, 6 (3–8) µg/mL to 7 (4–9) µg/mL, *p* = 0.43.

The highest quartile for the LV ventricular filling pressure (E/e’) was compared with the other three quartiles, respectively E/e’>9 vs E/e’<9. Seven patients had an E/e’>9 at baseline who completed follow-up. In these patients, no echocardiographic parameters improved nor worsened after anti-TNF therapy (Table 3).

Regression analyses showed no association between changes in inflammatory parameters, i.e., DAS28 and ESR, and changes in cardiac parameters, i.e., E/e’, E/A, GLS and NT-proBNP, between baseline and follow-up.

## 4. Discussion

To our knowledge, this is the first and largest study investigating the effect of first line anti-TNF therapy on the cardiac function in a Western RA cohort assessed with comprehensive echocardiography (including conventional, Speckle tracking and tissue Doppler) in combination with cardiac biomarkers. In contrast to what was expected, the echocardiographic results did not show improvement but importantly also showed no worsening of the cardiac function. Moreover, this study found a 23% decrease of NT-proBNP after six months of anti-TNF therapy, although this did not reach statistical significance.

Overall, diastolic function categorized in grades changed in only one case. The E/e’-ratio, a robust marker for predicting LV filling pressures and indirectly diastolic (dys)function [31], also did not show a significant change at follow–up compared to baseline, not even when comparing the highest quartile with the other quartiles. Examination of the systolic ventricular function was done using the LVEF and the more sensitive GLS to pick up more subtle changes. However, again, no improvement nor deterioration was observed. These unexpected results can be explained by the following causes. First, this could have been due to the low prevalence of cardiac dysfunction at baseline as only three (7%) patients had diastolic dysfunction. Therefore, the study may have been underpowered to show the improvement of the cardiac function. According to the literature, RA patients are more likely to have echocardiographic parameters of diastolic dysfunction in comparison to the general population [8,32,33]. Unexpectedly, our population had a lower-than-expected prevalence of diastolic dysfunction. This is explained because in our study diastolic function was assessed according to the updated 2016 ASE/EACVI grading criteria [29]. This grading algorithm applies several echocardiographic parameters and is more critical than previous grading algorithms of the 2009 ASE/EACVI criteria [34] and the Redfield criteria [35]. The primary goal of the 2016 ASE/EACVI update was to simplify the approach and hence increase the utility of the guidelines in daily clinical practice. However, recent studies also demonstrate a higher specificity and a lower sensitivity of the 2016 ESE/EACVI criteria resulting in a lower overall prevalence of diastolic dysfunction compared to the 2009 ASE/EACVI criteria. In addition, the 2016 ASE/EACVI criteria shows superiority over the 2009 criteria in predicting mortality, myocardial infarction and heart failure [36] and importantly in predicting increased left ventricular pressure measured with invasive assessment [37], thus making it currently the best available non-invasive diastolic dysfunction assessment tool. In comparison, previous studies mostly used the Redfield criteria, which is primary focused on the E/A ratio and the E-wave deceleration time (DT). When applying the 2009 ASE/EACVI and Redfield criteria in our cohort, the prevalence of diastolic dysfunction at baseline is considerably higher with respectively fifteen (33%) and eleven patients (23%). However, again, no relevant changes were observed at follow-up.

Second, this population had a relatively short disease duration and a relatively low disease activity compared to other studies, demonstrating an increased prevalence of cardiac dysfunction in RA patients compared to healthy subjects [8,38]. In addition, our population was relatively cardiac healthy as patients were relatively young and had low prevalence of cardiovascular comorbidities, and were thus less prone to the development of cardiac dysfunction. This is also suggested by the low H2FPEF score calculated for this cohort. The H2FPEF score is a method to assess the risk of the presence of heart failure with preserved ejection fraction (HFpEF) in patients with dyspnea and comprises a major risk factor for developing diastolic dysfunction. A large majority of the subjects (94%) had a H2FPEF score ranging from 0–2 and thus, a low a priori chance for diastolic dysfunction. This is also confirmed by the low serum NT-proBNP at baseline, indicating a normal cardiac wall tension. Another possibility could be due to the limitation of echocardiography in the assessment of diastolic function and the lacking ability to detect subtle diastolic changes. Possibly more sensitive assessment methods, such as exercise echocardiography [39], exercise right heart catheterization [40] or cardiac magnetic resonance imaging (MRI) [41], could help detect mild diastolic dysfunction and subtle diastolic changes. However, GLS analysis, a sensitive assessment method for the systolic dysfunction, did not show any alteration of the cardiac function. Furthermore, a large number of the patients used corticosteroids prior to the start of anti-TNF therapy, thereby affecting the anti-inflammatory effect of anti-TNF. Importantly, we found that that anti-TNF therapy had no detrimental effect on the cardiac function in patients with normal and relatively mild cardiac dysfunction.

Interestingly, studies assessing the effect of biologic agents with modes of action other than TNF-blockade show more evident ameliorating effects on the cardiac function. Several studies conducted by Ikonimidos et al. investigating the effect of anti-interleukin (IL)-1 therapy (anakinra) showed a significant improvement in cardiac function assessed with echocardiography [38,42,43]. Studies assessing the effect of anti-IL-6 (tocilizumab) on cardiac function assessed with cardiac MRI also showed an ameliorating effect on the diastolic function [44,45]. A possible explanation for this could be that IL-1 is produced earlier in the cytokine cascade and is the triggering factor of several cytokines including IL-6 and TNF-a [46]. Thus, theoretically, its inhibition may be more effective in controlling inflammation and thus improving LV function than inhibition of the later-released TNF-a. Whether anti-IL-1 and/or anti-IL-6 therapy have indeed more pronounced effects on cardiac function then anti-TNF is not known, as direct comparative studies have not been conducted. NT-proBNP is a predictor of CV mortality and morbidity in patients with or without a history of CVD and RA patients [47,48,49] and in our study, a 23% NT-pro BNP decrease after 6 months anti-TNF treatment was observed. This is in line with the findings of Kotyla et al., and Peters et al., where infliximab treatment or adalimumab treatment during 4 months led to comparable decreases of serum NT-proBNP in RA patients [22,50]. There are a few explanations for this phenomenon. Firstly, reduction of inflammation could have led to reduced ventricular stress. This is confirmed by the literature describing that inflammation contributes to arterial stiffening and consecutively increases ventricular load [51,52]. Secondly, low grade inflammation of cardiac tissue can lead to a stress response of the myocardium, leading to increased NT-proBNP production. This could explain the overall relationship between NT-proBNP and systemic inflammation [53,54]. However, a direct effect of anti-TNF on the production or secretion of NT-proBNP cannot be ruled out.

A major strength of this study is the comprehensive and prospective approach in the assessment of cardiac function with the use of conventional Doppler and Speckle tracking echocardiography in combination with cardiac biomarkers. Therefore, this study was able to determine the different outcomes assessing the cardiac function. The main limitation of this study was, as the prevalence of cardiac dysfunction was unexpectedly low, the rather limited sample size.

## 5. Conclusions

In conclusion, echocardiography showed no improvement nor deterioration of anti-TNF treatment on the cardiac function in RA patients with a low prevalence of cardiac dysfunction. However, NT-proBNP decreased 23% after anti-TNF treatment, which might suggest subtle improvement of the cardiac function.

## Figures and Tables

**Table 1:** Baseline characteristics.

Patient Characteristics	*n* = 47
Age, years	57 (±11)
Gender, female (*n*, %)	32 (68%)
RA disease duration, years	5 (2–19)
BMI, kg/m^2^	26.0 (±4.5)
History of vascular disease (*n*, %)	1 (2%)
Hypertension (*n*, %)	4 (9%)
Systolic blood pressure, mmHg	130 (±15)
Diastolic blood pressure, mmHg	80 (±10)
Hypercholesterolemia (*n*, %)	3 (6%)
Diabetes mellitus type 2 (*n*, %)	1 (2%)
Smoking (*n*, %)	13 (29%)
Rheumatoid factor positive (*n*, %)	33 (66%)
Anti-cyclic citrullinated protein positive (*n*, %)	32 (68%)
LDL, mmol/L	2.93 (±0.90)
HDL, mmol/L	1.57 (±0.50)
**Medication**	
Current oral corticosteroid use (*n*, %)	13 (28%)
Intra-muscular/intra-articular corticosteroid in the past 3 months (*n*, %)	9 (19%)
csDMARD use	43 (92%)
Methotrexate (with or without other csDMARD(s))	33 (70%)
Other csDMARD(s)	10 (21%)
Biological therapy (*n*, %)	
Etanercept	16 (34%)
Adalimumab	23 (49%)
Certolizumab	7 (15%)
Golimumab	1 (2%)
**Disease activity parameters**	
DAS28	4.44 (±1.23)
CRP, mmol/L	6.3 (3.1–20.0)
ESR, mm/h	22.5 (9.0–42.5)
Health assessment questionnaire (HAQ)	1.25 (0.75–1.50)
**Cardiac parameters**	
E/e’ ratio (*n* = 46)	7.9 (6.6–9.0)
E/A ratio (*n* = 46)	1.1 (±0.36)
Deceleration time, ms (*n* = 43)	0.22 (±0.06)
LA volume index, mL/m^2^ (*n* = 33)	28.40 (±7.53)
LV ejection fraction, % (*n* = 29)	63.1 (±8.8)
GLS (*n* = 44)	−19.8 (±3.5)
Impaired GLS (>−17%) (*n*, %) (*n* = 44)	6 (14%)
LV mass index, g/m^2^ (*n* = 42)	68.46 (±17.97)
Diastolic LV dysfunction (*n*, %) (*n* = 46)	
No diastolic dysfunction	38 (83%)
grade I	2 (4%)
grade II	1 (2%)
grade III	0 (0%)
indeterminate	5 (11%)
Systolic LV dysfunction (*n*, %) (*n* = 29)	2 (7%)
Aortic valve stenosis (*n*, %) (*n* = 46)	0 (0%)
Aortic valve regurgitation (*n*, %) (*n*=46)	6 (13%)
mild	6 (13%)
moderate	0 (0%)
severe	0 (0%)
Mitral valve stenosis (*n*, %) (*n* = 46)	0 (0%)
Mitral valve regurgitation (*n*, %) (*n* = 45)	18 (38%)
mild	18 (38%)
moderate	0 (%)
severe	0 (0%)
H2FPEF score	
0–1 points	32 (70%)
2–4 points	15 (30%)
5–6 points	0 (0%)
**Cardiac biomarkers**	
Troponin-T, µg/mL	6 (3–8)
NT-proBNP, ng/L	88.6 (47.0–142.0)

Values are displayed as mean ± standard deviation (SD), median (IQR) or frequencies with corresponding percentages (%). RA = rheumatoid arthritis, BMI = body mass index, LDL = low density lipoprotein, HDL = high density lipoprotein, csDMARD = conventional synthetic Disease Modifying Anti Rheumatic Drug(s), DAS28 = disease activity score-28, CRP = C-reactive protein, ESR = erythrocyte sedimentation rate, LA = left atrial, LV = left ventricular, GLS = global longitudinal strain, NT-ProBNP = N-terminal pro hormone-brain natriuretic peptide.

**Table 2:** Effect of anti-TNF on the cardiac parameters and disease activity parameters.

	Baseline (*n* = 47)	Follow-Up (*n* = 33)	*p*-Value
Cardiac parameters			
E/e’	7.9 (6.6–9.0)	7.7 (7.1–9.1)	0.17
E/A	1.1 (±0.4)	1.1 (±0.4)	0.94
Deceleration time, sec	0.22 (±0.06)	0.22 (±0.04)	0.44
LA volume index, mL/m^2^	28.4 (±7.5)	30.3 (±5.4)	0.21
LV ejection fraction, %	63.0 (±8.7)	62.0 (±7.9)	0.097
GLS	−19.8 (±3.5)	−19.9 (±2.6)	0.79
LV mass index, gram/m^2^	68.5 (±18.0)	73.0 (±22.4)	0.66
Troponin-T, µg/mL	6 (3–8)	7 (4–9)	0.43
NT-proBNP, ng/L	89 (47–142)	69 (42–155)	0.10
**Disease activity parameters**			
DAS28	4.44 (±1.23)	2.72 ± 1.23	<0.001 *
CRP, mg/L	6.3 (3.1–20.0)	2.6 (0.8–7.6)	0.009 *
ESR, mm/h	23 (9.0–43)	8 (5–15)	<0.001 *
HAQ	1.3 (0.8–1.5)	0.5 (0.0–1.3)	0.001 *

Values are displayed as mean ± standard deviation (SD), median (IQR) or frequencies with corresponding percentages (%). LA = left atrial, LV = left ventricular, GLS = global longitudinal strain, NT-proBNP = N-terminal pro hormone-brain natriuretic peptide, DAS28 = disease activity score-28, CRP = C-reactive protein, ESR = erythrocyte sedimentation rate, HAQ = health assessment questionnaire. * Significance level of *p* ≤ 0.05.

**Table 3:** Effect of anti-TNF in patients in the highest E/e’ quartile (E/e’ > 9).

E/e’ > 9 (*n*)	Baseline	Follow-Up	*p*-Value
E/A (6)	1.0 (±0.4)	1.0 (±0.3)	0.97
E/e’ (7)	11.8 (±3.0)	10.9 (±4.2)	0.34
deceleration time, sec (4)	0.23 (±0.06)	0.26 (±0.05)	0.34
ejection fraction (4)	62.0 (±7.4)	62.2 (±9.5)	0.97
LV mass index, gram/m^2^ (5)	83.0 (±26.1)	100.6 (54.1)	0.28
LA volume index, mL/m^2^ (4)	36.6 (±3.8)	36.6 (±3.8)	0.87
GLS (6)	−18.0 (±3.5)	−18.2 (±2.7)	0.90

Values are displayed as mean ± standard deviation (SD) or median (IQR). LA = left atrial, LV = left ventricular, GLS = global longitudinal strain.
